# Editorial: Mental health and suicide prevention in sensory impairment populations

**DOI:** 10.3389/fnhum.2026.1814341

**Published:** 2026-03-24

**Authors:** Sabrina Anne Jacob

**Affiliations:** Jeffrey Cheah School of Medicine and Health Sciences, Monash University Malaysia, Subang Jaya, Malaysia

**Keywords:** culture, hearing impairment, mental health, sensory impairment, visual impairment

People with sensory impairment (SI) represent a large and rapidly growing segment of the global population. The World Health Organization (WHO) has reported that globally approximately 466 million people have hearing impairment (HI), while 2.2 billion have vision impairment (VI). The prevalence of both are on an upward trajectory with estimates that by 2050, over 700 million will have disabling hearing loss due to aging and demographic shifts (World Health Organization, 2018, 2019b).

Sensory impairment is linked with markedly elevated risks of adverse mental health outcomes, including anxiety, depression, and suicide, compared to those without sensory deficits (Simning et al., 2019; Kvam et al., 2006; Witte and Kuzel, 2000; Hashemi et al., 2024; Shoham et al., 2019; Khurana et al., 2021). Despite this, significant gaps remain in the evidence regarding prevalence across different populations and age groups, help-seeking behavior and healthcare access, as well as risks and protective factors. This Research Topic brings together diverse empirical contributions that deepen understanding of the impact of SI on mental health.

Two studies explored the link between depressive symptoms and HI, using longitudinal survey evidence. Sun et al. drew on the 8th wave of the Chinese Longitudinal Healthy Longevity Survey (CLHLS) which was run from 2017 to 2018 and involved more than 5,000 adults aged ≥65; where researchers sought to explore the relationship between depressive symptoms, psychological wellbeing, and HI. Findings not only reveal the direct negative effect of auditory loss on both depressive symptoms and psychological wellbeing, but also an indirect effect on depressive symptoms which is mediated through diminished psychological adaptability. The authors underline the significant protective role that psychological wellbeing can play in alleviating the impact of HI on depressive symptoms and call for a deeper understanding of the underlying mechanisms and pathways to develop more targeted interventions for this population.

Using data from close to 3,000 individuals aged ≥45 who were involved in the China Health and Retirement Longitudinal Study (CHARLS), Liu et al. developed and validated a nomogram to predict the risk between HI and depressive symptoms in middle- and older-aged adults. This tool would assist in risk stratification and early identification of individuals at higher risk for depression. The study revealed that more than 40% of this population experienced depressive symptoms, which markedly exceeded global prevalence estimates of 10–20%.

This aligns with the evidence from a nationwide cohort study by Wang et al. which also drew evidence from the CHARLS database, and involved more than 3,000 middle- and older-aged adults. Analysis demonstrated that increases in depressive symptom scores resulted in an increased risk of new-onset dual SI, with authors highlighting the importance of close monitoring of depressive symptoms to detect at-risk individuals. These observations also raise the question of the possible bidirectional relationship between depression and SI, suggesting possible shared biological and psychosocial pathways, and thus the need for further longitudinal investigations to determine causal mechanisms.

Two other studies looked at the link between affective disorders and VI. A cross-sectional study by Niu et al. involving patients aged ≥65 with cataracts—a major cause for VI—found that those with economic hardship, comorbid hearing disorders, and poor self-reported health status, were more likely to develop depressive symptoms. These findings underscore the need for more targeted public health interventions to raise awareness about the importance of early risk assessment and health screening, as well as the need for changes to government policies to help alleviate the financial burden faced by this population.

While other studies focused on depressive symptoms, Yao et al. focused on symptoms of anxiety in adults aged ≥65, also drawing data from close to 12,000 participants from the CLHLS database. Individuals with VI were found to have significantly higher levels of anxiety, with binary logistic regression modeling indicating that it was a significant predictor for anxiety symptoms in older adults. The model also found that poorer sleep quality, a prominent experience of older adults with VI, could also exacerbate these symptoms.

## Implications for practice, policy, and future research

All five studies were rooted in Chinese populations, particularly in older adults, highlighting the need for more globally representative research, especially in lower socioeconomic countries where the impact on mental health is even more pronounced (Liu et al.). While most countries have or are approaching an aging population (World Health Organisation, 2015)—who have an increased risk of SI (World Health Organization, 2022, 2019a)—evidence points to high prevalence rates even in middle-aged adults, warranting more attention to this age group as well.

It was observed that suicide was not assessed in any of these papers and is rarely done so in other studies. Culture has a significant impact on help-seeking behavior, with many traditional or collectivist cultures often focusing on physical rather than mental health (Liu et al.) (Zhou et al., 2022). Stigma related to mental health especially suicide, is also frequently seen as a barrier to seeking care (Schomerus and Angermeyer, 2008; Wyllie et al., 2025), thus hindering early detection, diagnosis, and more importantly, management. Indeed, a common underlying thread in the included studies is the importance of early identification and risk stratification. As such, the importance of culture cannot be overlooked.

Similarly, the impact of mental health on SI should be examined as studies show it can exacerbate these conditions, rather than just being a result of them (Shin et al., 2021; Jiang et al., 2025) (Wang et al.). However, future studies should move beyond examining prevalence rates and risk factors. The study by Sun et al. revealed the impact of SI on psychological wellbeing as well, and as such more qualitative work to explore the lived experiences of patients should be undertaken to ascertain the real impact on psychological wellbeing, life satisfaction, and quality of life, as all have a significant bearing on mental health. This also highlights the importance of adopting a multifaceted approach when treating people with SI, integrating care for sensory issues with larger health and wellness programs focusing on risk factors that impact mental health such as diet, sleep, social support etc. Indeed, further studies should explore the role of diet and certain food types in mitigating mental health risks in this population (Yao et al.; Niu et al.).

Healthcare professionals must also be cognizant of the fact that there is no “one-size fits all” when it comes to interventions/programmes. Indeed, research has shown different risk factors and barriers to seeking help based on socioeconomic backgrounds (Ravesloot et al., 2014). Thus, for any intervention to be truly meaningful and effective, a community-based participatory approach must be adopted, where those affected by the outcomes of these interventions/programmes are included as active partners through co-design (Johnson, 2023). This will enable more tailored interventions, enhance patient engagement, and foster a sense of ownership over the programmes developed (Viswanathan et al., 2004).
